# A Consensus Statement for Ecological Medicine: Moving Toward Connection-Based Medicine

**DOI:** 10.1007/s10393-025-01757-3

**Published:** 2025-10-25

**Authors:** Michael Makhinson, Landon Pollack, Ronan Hallowell, Conor H. Murray, Jay E. Maddock, Stephanie Michael Stewart, Avik Basu, David King, Helena Hansen, Ecological Medicine Working Group, Allison C. Alberts, Brian Anderson, Kate Armstrong, James C. Bassett, Francis J. Baumont de Oliveira, Jamie Beachy, Manijeh Berenji, Anya K. Bershad, Joanna E. Bettman, Daniel T. Blumstein, Lindsay Branham, Matthew H. E. M. Browning, Taimie L. Bryant, Rebecca M. Calisi Rodriguez, Yosuke Chikamoto, Benjamin Collins, Ziva D. Cooper, Dana Cuff, Cynthia Davis, Katrina F. DeBonis, Somayeh Dodge, Lynette A. Hart, Marco Iacoboni, Celina M. De Leon, Aubrey H. Fine, Lisa R. Fortuna, Sam Gandy, Nancy Gee, Diana S. Grigsby-Toussaint, Charles S. Grob, Suma Jacob, Jessica K. Jeffrey, Laura H. Kahn, Nurit D. Katz, Michael Kaufmann, Heather Kuiper, Beatriz C. Labate, Sheila Laffey, Tieraona Low Dog, Todd Lynch, Olivia McAnirlin, Joseph McCowan, Daphne Miller, Barbara Natterson-Horowitz, Alessandro Ossola, Teresa L. Penbrooke, Patricia Pendry, Michelle V. Porche, Jessica D. Pratt, Elena Rios, Sylvie Rokab, Jose Sanchez, Jack Saul, Felix E. Schweizer, Sue Sisley, Martyna Skalna, Wendelin Slusser, Olga Solomon, Melissa Sundermann, Sujit Thomas, Karen Waconda Lewis, Rosalind Watts, Jennifer Wolch, Lee Zasloff

**Affiliations:** 1 Department of Psychiatry and Biobehavioral Sciences, David Geffen School of Medicine, and the Jane and Terry Semel Institute for Neuroscience and Human Behavior, University of California, Los Angeles, Los Angeles, CA USA; 2 Department of Psychiatry, Harbor-UCLA Medical Center, 1000 West Carson Street, Box 8, Torrance, CA 90502 USA; 3 Department of Medical Education, Keck School of Medicine of the University of Southern California, Los Angeles, CA USA; 4 School of Public Health, Texas A&M University, College Station, TX USA; 5 Department of Psychiatry, Charles R. Drew University of Medicine and Science, Los Angeles, CA USA; 6 School for Environment and Sustainability, University of Michigan, Ann Arbor, MI USA; 7 Department of Psychiatry and Behavioral Sciences, Stanford University School of Medicine, Palo Alto, CA USA; 8 Center for Social Medicine and Humanities, University of California Semel Institute, University of California, Los Angeles, Los Angeles, CA USA

**Keywords:** ecological medicine, nature-based interventions, ecotherapy, social prescribing, nature contact, nature and health

## Abstract

Mounting evidence across multiple disciplines supports the health benefits of connection to nature. Although this trend suggests that the human-nature relationship is integral to health, its importance is often overlooked in clinical practice due, in part, to lack of consensus on its scope, limits, and terminology. To fill a needed gap, we developed a consensus statement on an inter-connectivity based view of health termed Ecological Medicine. The study recruited an expert working group and used modified Delphi technique and focus groups. The Ecological Medicine Working Group was directed toward Ecological Medicine consensus goals that included: (1) a consensus definition and framework, (2) priorities for practice, research, education, and policy, and (3) Ecological Medicine’s implications. A consensus definition and framework for Ecological Medicine was reached, focusing on the importance of human inter-connections (to self, others, non-human species, and natural environment) in informing health understanding. Ecological Medicine suggests that healthcare should shift toward inter-connectivity, relationality, and health practices involving connection-based interventions, especially nature-based interventions. This framework may benefit research, practice, education, policy and other domains of healthcare by focusing on the importance and benefits of connectivity-based health interventions and on the inseparability of human health and planetary health.

## Introduction

Despite unprecedented advances in medical knowledge, global health faces concerning trends across multiple health domains, including increasing anxiety and depression, mounting chronic disease prevalence, and widening health disparities. Societal costs are severe: mental health disorders could cost the world economy $6 trillion annually by 2030 (World Health Organization, 2022), and the global cost of chronic disease may reach $47 trillion by 2030 (Hacker, 2024).

The standard biomedical model of practice has historically been focused primarily on the individual; recent approaches have questioned and expanded this focus. Approaches such as the bio-psycho-social model (Engel, 1977), holistic medicine (Gordon, 1982), integrative medicine (Maizes et al., 2009), and lifestyle medicine (Lippman et al., 2024) have broadened ideas of medical functioning and wellness. There is increasing recognition that inter-connections are integral to health and wellbeing; the “epidemic of loneliness” (Office of the Surgeon General, OSG 2023) has been cited as a national health concern. Beyond human–human relationships, research suggests the importance of broader connections, particularly between humans and nature. Various forms of nature connection are suggested to improve human health. Examples include improved post-operative and hospital recovery with views of nature or presence of hospital greenspaces (Ulrich, 1984; Sherman et al., 2005), improved mental and physical health with interactions with gardens or greenspaces (Soga et al., 2016; Dean et al., 2018; Zhao et al., 2022), decreased stress with shinrin-yoku (forest bathing) (Park et al., 2010), improved physical and emotional health with companion animals or animal assisted therapy (Johnson, 2013; Jennings et al., 2021), and improved stress markers with passive nature contact (e.g., nature images, virtual reality, and listening to birdsong) (Qi et al., 2022; Schebella et al., 2019). Improved child cognition as measured by attention and memory tasks has been linked to school greenspace (Vella-Brodrick and Gilowska, 2022). Biophilic design-informed urban planning has been demonstrated to confer a variety of health benefits, such as improved cardiovascular risk profile in one study (Makram et al., 2023) and improvement in multiple health outcomes preferentially in low socioeconomic status groups in another study (Rigolon et al., 2021). There are explanatory theories of nature contact, including Attention Restoration Theory (ART) (Berman et al., 2008), which proposes that time in nature improves cognitive functions such as attention and concentration, and Stress Recovery Theory (SRT) (Ulrich et al., 1991), which proposes that nature reduces negative mental and physiological consequences of stress. However, most people spend little time in natural environments, with time in nature decreasing and time spent on digital devices increasing (Kellert et al., 2017).

Adoption of a connectivity-based health framework which expands the current biomedical model to multiple levels of inter-connectivity and recognizes profound interdependencies between human beings, other living elements of nature, and the natural environment, would be beneficial to human health and to the health of the biosphere. To date, there is no consensus framework in the literature to facilitate the adoption of such a connectivity-focused health paradigm. Ecological Medicine could provide such a framework. To address these gaps, this study aimed to: (1) recruit a diverse working group of inter-disciplinary experts to develop a consensus framework of a connectivity-informed healthcare view, Ecological Medicine, (2) use a modified Delphi method (Brown, 1968) to define Ecological Medicine and its priorities in research, clinical practice, education, and policy, and (3) use focus sub-groups of the “Ecological Medicine Working Group” to understand the implications of adopting the Ecological Medicine framework.

## Methods

**Figure 1:**
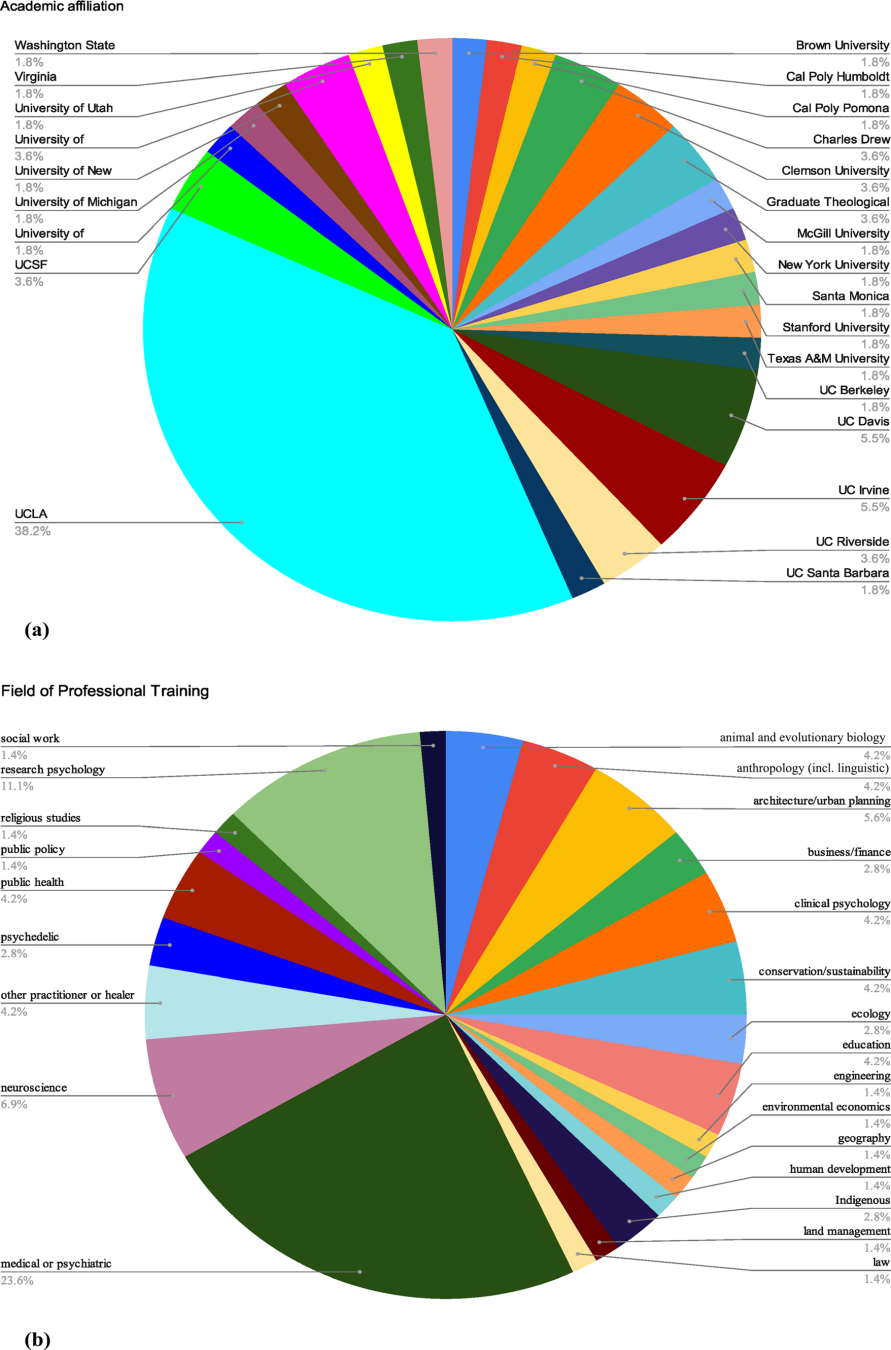
demonstrates the professional characteristics of the 73 members of the Ecological Medicine Working Group. Figure 1a presents the primary academic affiliation of the members of the Working Group that identified as academically affiliated (57 out of 73). Figure 1b presents the primary professional fields identified by the Working Group.

A mixed methods process was used to achieve consensus on the definition and priorities of Ecological Medicine. An IRB exemption was received prior to initiation. Initial steps utilized the modified Delphi method (Brown, 1968), and the final step used focus groups. First, a scoping narrative literature review was completed in October 2024. Co-authors identified relevant references to Ecological Medicine and related concepts, without specific inclusion or exclusion criteria. Indices searched in the literature review included MEDLINE and Google Scholar, inclusive of all years. The review was submitted as a separate manuscript (not yet published) and used to inform subsequent steps. Next, a multi-disciplinary team of academic researchers, practitioners, educators and advocates were chosen by the principal authors (*N* = 73). Selection was decided by consensus, expert recommendations, professional relationships, and need for discipline diversity. The principal authors agreed on the importance of including Indigenous scholars and practitioners. The group formed the Ecological Medicine Working Group (henceforth shortened to “Working Group”). 4/73 (5.5%) identified as Indigenous researchers, or practitioners. The group represented a diverse set of professional fields, shown in Figure 1. Figure 1a shows the group’s affiliations: 55/73 (75.3%) were academically affiliated, with the largest affiliation being UCLA (21/73 [28.8%]). The group was geographically diverse, residing in 15 U.S. states and 5 countries, with the largest contingent (48/73 [65.8%]) from California. A pre-meeting survey was distributed in October 2024 to frame initial discussion of Ecological Medicine’s foundational priorities. Results were analyzed via Qualtrics, shown in Table 1. In November 2024, the Working Group met in-person to discuss pre-meeting survey results and to refine the definition of Ecological Medicine. The group was divided into themed focus sub-groups to aid in consensus prioritizations. In January and February, 2025, the Working Group met twice virtually to further refine consensus and plan for future steps. Between February and June 2025, 6 virtual meetings were held with each themed sub-group (research, clinical practice, curriculum, and community engagement).

**Table 1:** Survey results from the Ecological Medicine Working group.

Consensus priority for ecological medicine	Mean prioritization rating(± SD)
The benefits (and potential disbenefits) of increased contact with nature, at the individual, community, and population level, to all aspects of human health. This includes physical health and well-being as well as a broad scope of mental health (encompassing topics such as psychological well-being and stress response, positive behavioral changes, mental illness recovery, addiction, and neurodiversity)	Mean 7.99 SD (± 1.55)
Indigenous ways of knowing and traditional ecological knowledge. Aspects of this idea include: a) development of non-colonial and regenerative practices, b) acquisition of knowledge and practices in ways that are not transactional but rather relational, reparative, and reciprocal according to the terms set by the Indigenous partner and community, and c) meaningful inclusion of Indigenous people and communities in design and decision-making, and commitment to the care for Indigenous communities’ needs	Mean 7.59 SD (± 1.62)
The potential adverse consequences to human health (and mechanisms of action) at the individual, community and population level, of lack of nature access, lack of sense of connection to nature, local and macro environmental destruction, and environmental injustice	Mean 7.56 SD (± 1.82)
Mechanisms by which contact with nature produces health benefits. This includes critical assessment of current ideas such as the biophilia, attention restoration, stress recovery theories, and feelings of awe. Also included, detailed and critical examination of which geographic and sensory elements of nature environments confer benefits, along with which contact modalities (e.g., physical immersion, specific sensory exposures, XR exposure)	Mean 7.50 SD (± 1.51)
How nature should be integrated into the existing medical system, including dimensions of access, contact, exposure, and justice. This includes the use of nature prescriptions, understanding patient selection, indications, training, clinical research, and health care system impacts	Mean 7.18 SD (± 1.68)
Understanding the interrelationship between human, animal and environmental sustainability. This understanding entails shifting from a human-centric, exceptionalist view to a more interspecies perspective	Mean 7.15 SD (± 2.08)
The potential reciprocal benefit to human health and to health of the ecosphere of adopting a global view of nature and all of its inter-species relationships, such as the views adopted by Planetary Health, One Health, and Conservation Medicine	Mean 7.09 SD (± 2.00)
How best to incorporate principles of equity and justice into contact with nature, using principles of distributive, procedural, corrective, recognitional, and restorative justice	Mean 7.00 SD (± 1.69)
The incorporation of contact with nature into architecture, design, and urban planning. This includes goals of: a) benefits of contact with the natural elements to individuals, organizations, and cities through nature-centered, sustainable design and planning, and b) optimization of outcomes such as health, environmental sustainability, and equity and justice	Mean 6.94 SD (± 1.90)
How to quantify the economic benefits of nature that are mediated through improved health and well-being, such that understanding how to value these benefits could help inform policy decisions	Mean 6.77 SD (± 1.72)
How nature is defined. Dimensions of this include: a) cross-cultural study of how nature is defined and understood, b) the variety of human understanding of the relationships and reciprocity surrounding nature, including plants, animals, ecosystems, and the biosphere, c) the conceptualization by legal systems of components of nature as legal entities with rights, and d) the different approaches to defining and studying nature by different disciplines	Mean 6.42 SD (± 1.86)
The relationship between contact with nature and spirituality. Aspects of this idea include understanding how spirituality and nature have been co-emergent historically, cross-cultural study of spirituality and nature conceptualization and connectedness, and best practices for inclusion of those with spiritual training in the discussion of nature and human well-being (such as eco-chaplains, eco-theologians, and shamans)	Mean 6.25 SD (± 2.14)
The impact and potential of contact with animals generally and animal-assisted services specifically on human health. This includes companion animal ownership/guardianship, physical therapy/treatment and mental health, incorporating animals, experiential education with animals and a broad range of indirect animal focused activities (bird watching, wildlife observation, beekeeping, etc.)	Mean 6.15 SD (± 2.11)
The impact of horticultural therapy on human health, focusing on cross-cultural practices	Mean 5.80 SD (± 1.67)

The group was asked the question: please read each core idea and rate the importance of its inclusion within the domain of Ecological Medicine (as you understand it) on a scale from 1 (LEAST important as a core focus) to 9 (MOST important as a core focus). A mean score (along with standard deviation) was calculated and presented in the table, with the priority items listed with highest score at the top (representing the highest consensus priority as rated by the group).

## Results

### Ecological Medicine: A Consensus Definition

The Working Group arrived at the following consensus definition:

Ecological Medicine is a conceptualization of health and well-being as an interdependency between human beings, other species of life on Earth, and the environment which supports them. Human health cannot be understood, examined, or improved fully without relationality, because life processes proceed in relation to other life processes and also in relation to their surrounding environments. In effect, a concept of a single organism, its individual health, and its functioning, in isolation, will lead to a grossly incomplete understanding of the larger, interconnected network.

The primary theme identified by the Working Group was inter-connectivity as the key concept needed to fill the gap toward an expanded view of health. Humans may be understood by several levels of inter-connectivity: (1) connection to self, (2) connections to other humans, (3) connections to animals, plants, and other living kingdoms, and (4) connections to natural and built environments. The group agreed that inter-connectivity is important in human life, and inter-connections both confer health benefits and may have protective effects against disease. Conversely, disconnection syndromes may impair health and increase risk of disease. Examples include the contribution of social disconnection to overall health and mortality (Na et al., 2023), influence of social relationships on health outcomes (Shartle et al., 2022; Christakis and Fowler, 2007; Kumar et al., 2021), health benefits of companion animals (Christakis and Fowler, 2007), negative public health consequences of nature disconnection (Makram et al., 2023; Rigolon et al., 2021) and positive health benefits of nature connection (Zhao et al., 2022; Park et al., 2010; Qi et al., 2022; Schebella et al., 2019). “Nature deficit disorder,” a growing societal trend, is not yet well-understood (Louv, 2008). Modernity embraces human exceptionalism, resulting in discounted importance of human inter-connections to self, other humans, other life and the environment. This manifests a narrow subset of health information which is largely incomplete. This informed the new paradigm of Ecological Medicine. Relationality, a related theme, will be discussed in a subsequent section.

Ecological Medicine aims to further understand individual health. Beyond traditional vital signs and biomarkers, as well as ecological (Li, 2017) and social determinants of health, the Working Group felt that Ecological Medicine could contribute additional health parameters called Ecological Medicine vital signs. Development of these vital signs was deemed an important subsequent step for future planning:

1. Quality and depth of relationships with self and other life: self-connection, social connections, connections with other forms of life, planetary stewardship.
2. Frequency and intensity of immersion in natural spaces, gardening, animal contact, and other nature-connecting activities.
3. Strength and character of belief systems about relationships with other life and the environment, including reciprocity (meaning the presence of a relationship that fosters mutual well-being)
4. Barriers to connection with self, other, or nature.

The goal of Ecological Medicine is to enhance understanding of human well-being by recognizing essential layers of inter-connectivity and understanding relationality; it is a way of thinking that prioritizes inter-connectivity over individualism. It emphasizes ecological system wholeness, with dynamic connectedness and interdependency of all of its parts, living and environmental. It recognizes that human health is inseparable from planetary health, suggesting particular attention to ecological sustainability as a pro-connective health behavior.

### Consensus of Priorities for Ecological Medicine

The Working Group reached consensus for priorities of the Ecological Medicine approach, including research, clinical practice, education, and policy. Table 1 presents the consensus priorities listed in order of strength of consensus, along with a mean score and standard deviation. The score represents the mean rating from 1 (least important as a priority) to 9 (most important).

These priorities suggest a starting point for a group of approaches, clinical practice and research agendas, educational curricula, and policy and advocacy goals that could unite under Ecological Medicine. Despite the hierarchical ratings, the Working Group agreed that all priorities were foundational and merited inclusion.

### What is different about the Ecological Medicine approach?

“We are like islands in the sea, separate on the surface but connected in the deep.”– William James

Recent approaches, including One Health, Planetary Health, and Conservation Medicine, have pushed beyond traditional health toward inter-disciplinary, non-anthropocentric views of health. For example, One Health suggests understanding human health and disease by understanding interconnections between humans, animals, microorganisms and the environment (Kahn, 2017). Planetary Health further emphasizes dynamic changes in the environment, including climate change, and resulting trans-species health changes and feedback (Seltenrich, 2018). Conservation medicine further emphasizes health consequences of climate change and increasing anthropogenic influences on environmental and infectious disease risk (Daszak et al., 2004). Ecological Medicine expands further by redefining what constitutes health, and by extension, what constitutes a “patient.” Ecological Medicine emphasizes relationality and connection as determinants of health and prioritizes examination of inter-connections over the individual in isolation.

**Figure 2:**
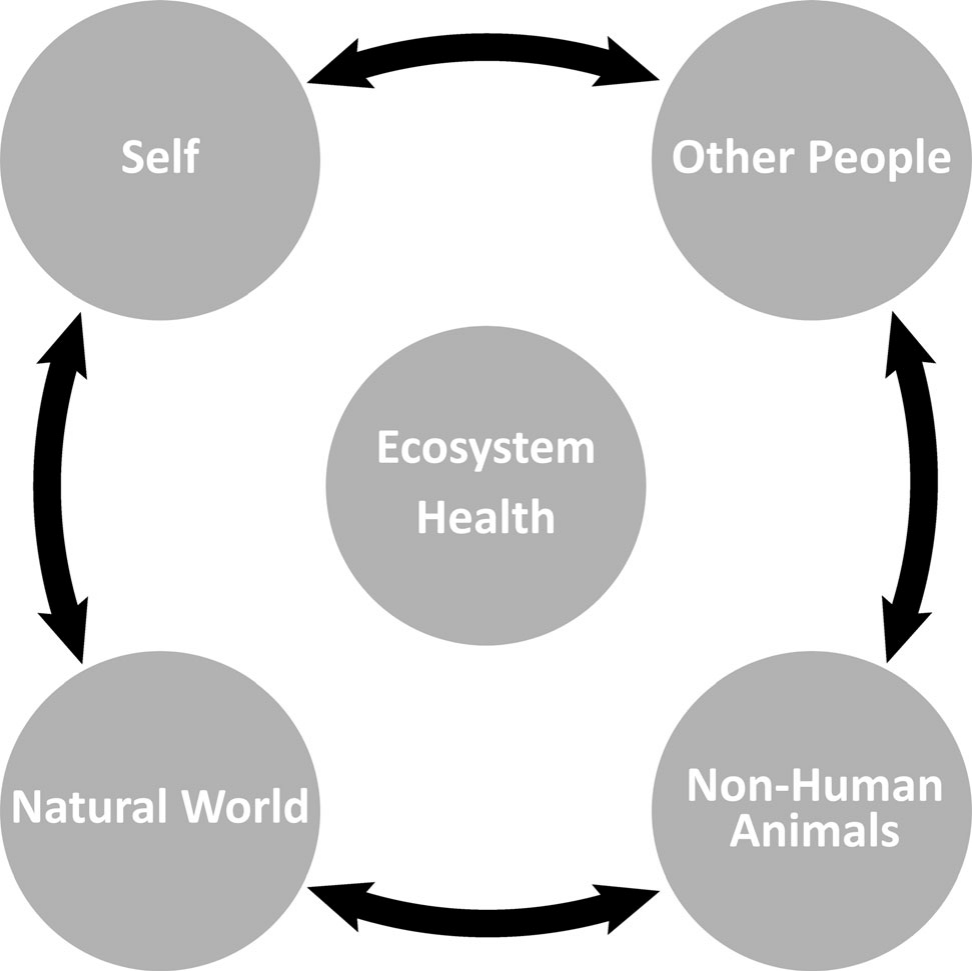
demonstrates the inter-connectedness of humans (including with self and others), non-human animals, and the natural world. The connections are dense and reciprocal, causing a deep inter-dependence. No element can be fully understood without understanding the total inter-connections, and all influence ecosystem health.

A growing number of fields and healthcare approaches have questioned the validity of examining human health in isolation. These include the aforementioned global health approaches, studies of health benefits of nature contact (see Frumkin et al., 2017 for a definition of nature contact), and renewed interest in many Indigenous views of health and traditional ecological knowledge. Relationality is a view that humans and other lives and processes are defined by their connections, with the connections being at least as important as the things that are being connected; the connections themselves are more instrumental in defining a system’s behavior than the individuals. Relationality views inter-connected relationships as reciprocal, with qualities of relationships (such as number, richness, and bi-directionality) defining the depth of relationality. The “patient,” would be a person with a biology, a lived experience, and social connection structure with an inter-dependent, reciprocal set of historical and current connections to (1) self, (2) family, (3) friends, (4) companion and other animals, (5) local plants and greenspaces, (6) microorganisms, (7) other frequently or infrequently visited natural environments, (8) relationship and reciprocity with the larger environment and planet, and (9) spiritual belief systems that aid in ascribing meaning to all of these relationships. The “patient” would be part of a vast, dynamic ecosystem with many inter-connected parts, demonstrated in Figure 2.

The concept of relationality has been examined in healthcare and biomedical research without a unifying framework. For example, in medical ethics, Herring noted that though relationality has been recognized between the disabled and caregivers, and between children and parents, it should be more widely understood as core to the process of living in all states of health. He wrote: “In a radical sense our relationships constitute ourselves and our identity. That is why relationships must be at the heart of an understanding of health” (Herring, 2016). A movement of relational medicine recognize the importance of interpersonal connection within the medical system, introducing concepts such as “clinician presence” (Brown-Johnson et al., 2019). Beyond medicine, Daniel Siegel, a psychiatrist and neuroscientist, has advocated for the importance of relationality in neurodevelopment, mental functioning, and conceptualizing mental disorders and their treatments. He has developed interpersonal neurobiology, which suggests that relational connections between minds are essential to understand human development and mental health. He wrote “a definition of the mind means that our mental lives emerge from beyond simply the brain in the head and involve the whole of the body; and mind also emerges within our relationships with people and the whole of the planet.” (Siegel and Drulis, 2023). Similarly, the psychiatrist Iain McGilchrist wrote: “I suggest that relationships are primary, more foundational than the things related: that the relationships don’t just ‘connect’ pre-existing things, but modify what we mean by the ‘things,’ which in turn modify everything else they are in relationship with.” Surveying research and clinical phenomenology of hemispheric laterality, he concluded that the right hemispheric processing mode (different from that of the left), provides a model of the world which is relational, aware of the whole, and non-reductionistic (McGilchrist, 2021). In studying human emotions, the psychologist Dacher Keltner has described awe as an emotion that generates increased connectivity to others, to nature, and to the mysteries of the world and existence (Monroy and Keltner, 2023). In the study of hormones, oxytocin has been tied to enhanced group cohesion, with increased trust, cooperation, and group synchrony (Patin et al., 2018).

Relational approaches have been adopted by many other scientific fields, including botany, environmental science, geography, public health, evolutionary biology, immunology, neuroscience, and physics, among others (Eyster et al., 2023). Listed are selected examples: (1) Suzanne Simard demonstrated extensive communication between trees, mediated in part by mycorrhizal networks (Gorzelak, 2015). These networks interact with other inter-tree networks to create complex adaptive systems, blurring the idea of trees as individual organisms. (2) Michael Levin conceptualized a cellular level of intelligence through the structure of bioelectric networks that unify cells toward a common goal. Following from this, cancer cells may be disconnected from surrounding cells in their inter-connected network identity and relationship to shared goals (Levin, 2025). (3) Nicholson and Dupre advocated for a process-focused biology, where biological concepts are best understood as the flow of processes that arise from the interdependent hierarchies of structure and function in organisms. For example, if colonies or symbiotes share processes, they suggest an expansion of the concept of individuals (Nicholson and Dupre, 2018). (4) Methot and Alizon proposed a more complex view of pathogen-host interactions, with no absolute pathogens but rather sets of ecological conditions and dynamic interactions which result in pathogenicity under some conditions but not others (Méthot and Alizon, 2014). (5) The physicist Carlo Rivelli suggested a variant approach to quantum mechanics termed relational quantum mechanics (RQM), in which a quantum system is only understood on the basis of the observation of its interactions: “the description of a system, in the end, is nothing other than a way of summarizing all the past interactions with it, and using them to predict the effect of future interactions.” RQM systems are solely defined by the relationships between elements of matter, including with the observer (Rovelli, 1996). (6) Lovelock and Margulis proposed the Gaia hypothesis, positing that the Earth is a self-regulating, complex system like a super-organism, in which organic life and inorganic parts of the planet interact and co-evolve in a self-maintaining way (Lovelock, 2003). Gaia acknowledged primacy of complex interactions and questioned the separation between individual organisms. Though never widely accepted, Gaia has influenced concepts such as Earth systems science and the Anthropocene era. In these examples, varied scientific fields recognize that omitting relationality in systems may preclude their complete understanding, missing recognition of emergent layers that could ultimately transform conceptualization.

Despite lack of adoption of relationality in healthcare and biomedical paradigms, it has long been central to many Indigenous systems of knowledge, research, and health. There is ample research on relational knowledge systems of diverse Indigenous nations, including how relationality influences Indigenous view of health, disease, and healing. Studying Indigenous health systems, McKivett and Paul noted that Australian First Nations place more importance on relationships with family, community, body and mind, spirit, ancestors, and land, with regard to healing and health sustenance. Also central is a belief that Earth is alive, with health sustenance dependent on right relationship with the Earth. In contrast, biomedical models are reductionistic, individual-focused, and human exceptionalist (McKivett and Paul, 2024). Researching a Mohawk community in Ontario, John and Castleden noted several themes in the community’s conception of health, including a holistic view of health inextricable from family relationships and community, importance of connection to the community, and centrality of relationships to the process of healing (John and Castleden, 2025). Redvers, in a knowledge sharing study of First Nations elders in Canada’s Northwest Territories, noted important differences between Indigenous and biomedical healthcare views (Redvers et al., 2024). These included: (1) primacy of law coming from Nature rather than from people, and health and healing come from connection to the land and to nature; (2) factors negatively affecting the health of Nature will affect the health of all species including humans, with paramount relevance to climate change and biodiversity loss; (3) products of Western science are not always compatible with Indigenous ways because of chemicals and the capitalist system that created them may adversely affect planetary health, and (4) recognition of deep inter-connectedness between people and all things of Nature (“the plants, rivers, lakes, wind, are all our relatives out there”)—anything that heals or harms one thing heals or harms the others. This expression of relationality and inter-connectedness of health offers insights to Ecological Medicine, while acknowledging that Indigenous people suffer disproportionate environmental and health harms (United States Environmental Protection Agency, 2025).

## Discussion

### Implications for Clinical Practice

Ecological Medicine’s focus on inter-connectivity and relationality has significant implications for key aspects of healthcare such as preventative health and disease management. Since nature contact improves health and reduces risk of multiple disease states, it should be incorporated into primary prevention practices and policy. Movements incorporating relational medicine, including social prescribing and nature-based interventions, should join other preventative measures such as healthy diet, exercise, seatbelt use, tobacco cessation, moderation of alcohol, and health screening. As primary prevention has added important diagnostic assessments, so should Ecological Medicine. Maddock and Razani argued that nature contact could follow an analogous path to physical activity as a health behavior, including: (1) creation of professional societies, (2) standardizing common measurements and research methodologies, (3) publishing expert guidelines, (4) creating journals and educational documents, (5) promoting adoption by government health agencies, and (6) creating reimbursement rules for clinicians (Maddock and Razani, 2024).

**Figure 3:**
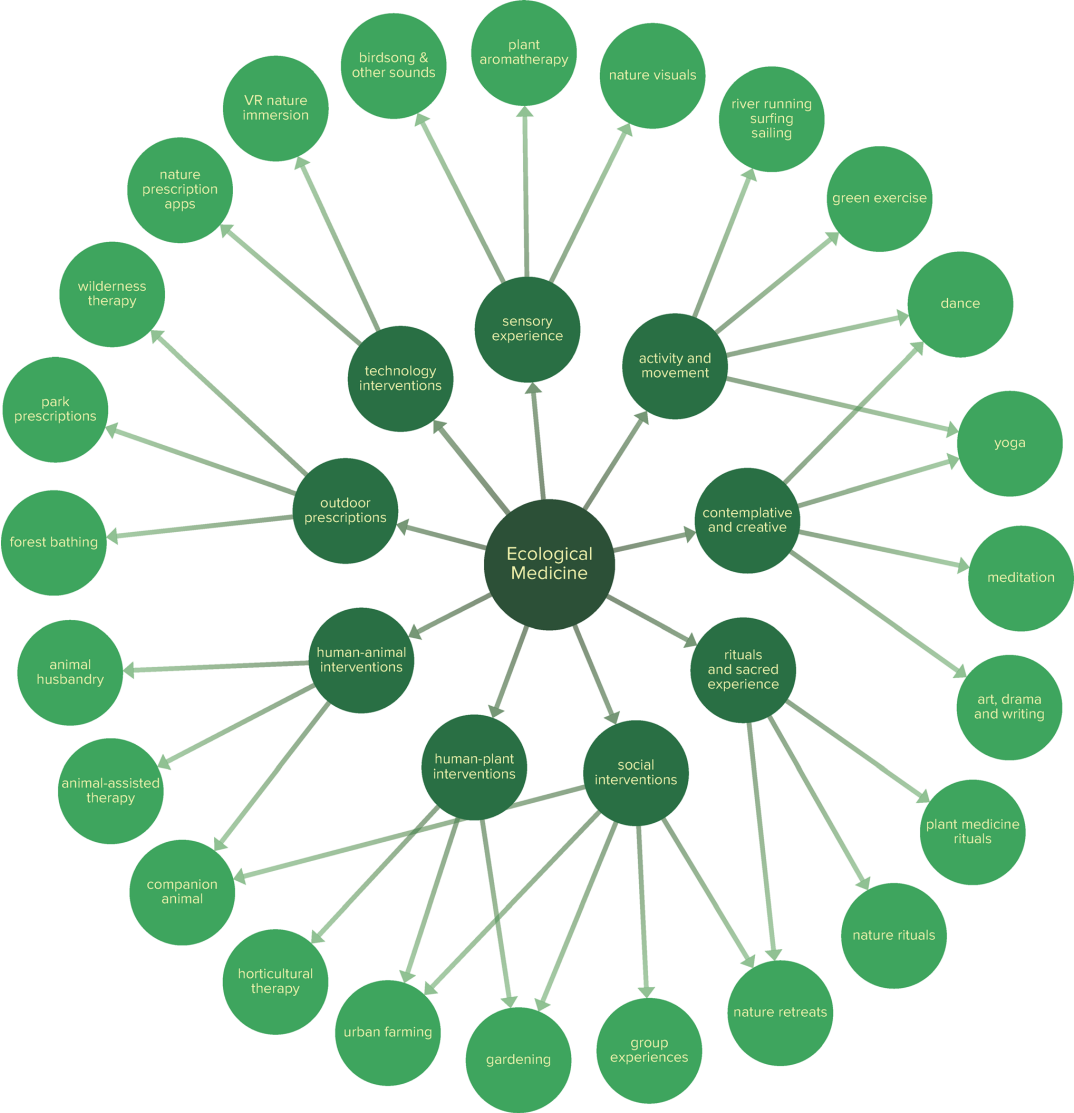
shows a sample of clinical interventions relevant to Ecological Medicine. All of these interventions serve to improve connection. Many are related to nature contact involving plants, animals, and the environment, while others foster connection with self and with others. They are grouped into categories of outdoor prescriptions, human-animal interventions, human-plant interventions, social interventions, rituals and sacred experience, contemplative and creative interventions, activity and movement, sensory experiences, and technology. Categories are not empirically validated and may be incomplete. Created using kumu.io.

Regarding health interventions, there are already a growing number of systems at levels ranging from ecologically-aware individual practitioners to community health systems that encourage nature prescriptions and national health systems that are instituting national programs for nature immersion (such as Japan and South Korea’s promotion of forest bathing). Nature prescriptions can take numerous forms, such as time in greenspaces (e.g., parks or backyards), companion animals and animal-assisted therapy, gardening and horticulture therapy, urban farming, immersive nature contact such as forest bathing, or less intensive contacts such as birdsong sounds, visuals of nature landscapes, or virtual reality (Nguyen et al., 2023). Alongside nature prescriptions, contemplative and artistic therapies and activities may encourage self-connection and integration, and group therapies and activities may encourage connection with others. Figure 3 presents a sample of clinical interventions consistent with the Ecological Medicine approach.

### Implications for Research

Research in Ecological Medicine is already fertile and growing, with many parallel disciplines working toward understanding relationships between humans, other species and the environment, while testing a variety of interventions. Disciplines include clinical medicine, environmental psychology, public health, ecology, anthropology, botany, sustainability science, geography, anthrozoology, plant medicine, forestry, architecture and urban planning, virtual reality, and many others. These fields are establishing the current foundations and evidence base for Ecological Medicine and for relational understating of humanity, health, and the biosphere. However, some factors may be limiting progress. One is funding: inadequate funding prioritization and recognition of the field by healthcare organizations, academic departments, foundations, and governments. Another limiting factor is the silo effect: research fields follow paths and methodologies in isolation from each other, without broad inter-disciplinary communication and collaboration. Consequences might be limited sharing of research ideas and methodologies, limited platforms toward advocacy for funding and prioritization by healthcare organizations, and limited impact and mindshare among investigators and academic departments. In contrast, a unified structure of Ecological Medicine could clarify and accelerate research priorities and methodologies, improve collaboration, improve funding advocacy and prioritization by healthcare organizations, and increase the academic profile of the field.

Clinical interventions in Ecological Medicine should be validated, with cost effectiveness demonstrated, resulting in adoption and integration into appropriate healthcare settings. Implementation research and health promotion would aid in engagement and adoption. Ultimately, meaningful adoption of Ecological Medicine into mainstream healthcare may require landmark studies, as have other paradigms such as cardiovascular disease prevention, diabetic treatments, cancer prevention, and women’s health. More large-scale, high-impact research studies will require acceleration of funding, advancement of trans-disciplinary collaboration, and increasing the scale of Ecological Medicine organizations.

### Additional Implications

Ecological Medicine should inform many other organizations and societal systems. Wide spectra of educational systems and curricula, including undergraduate education, medical education, and that of many non-medical clinical fields should include foundations of Ecological Medicine. The relationality of health and the biosphere is fundamental to understanding health from the human to the inter-species to the biospheric level, suggesting the value of its incorporation in clinical educational systems. Current educational paradigms, especially those in science and medicine, may have limitations, such as being influenced by compartmentalization of academic fields, a lack of inter-disciplinary bridging, and relative exclusion of fields that question human exceptionalism. Ecological Medicine may provide inter-disciplinary bridging and a focus on inter-connectivity and relationality.

Urban design and planning, such as parks, schools, hospitals, public spaces, and housing communities should incorporate biophilic design (Richardson and Butler, 2021). Urban design can intentionally guide structures, spaces, and communities toward nature connection, ultimately with goals of increased harmony with nature, sustainability and increased mental and physical health. Public health policy should also be informed by Ecological Medicine. Public policy campaigns at national and local levels have been successful in influencing health interventions such as vaccination, cancer screening, prenatal care, and tobacco cessation. Improved nature contact could also be a valuable policy goal. Relevant goals could include increasing equitable public access to natural spaces, and encouraging conservation and outdoor recreation. Healthcare economics studies may be helpful in demonstrating cost-efficient benefits of nature prescriptions. Related to public policy, disaster recovery may be another field where Ecological Medicine might be helpful. There have been increasingly frequent extreme climate events and disasters: events in 2024–2025 included the Los Angeles wildfires, Haji pilgrimage deaths in Mecca, flooding from Hurricane Helene in North Carolina and Tennessee, cyclones in Africa, India, and Bangladesh, and flooding in Spain and Brazil. While more research is needed, preliminary investigation of nature-based interventions in climate disaster recovery (Block et al., 2019; Hartwell et al., 2023) support an Ecological Medicine approach to disaster recovery where physical infrastructure as well as psychological health of affected communities are rebuilt through an ecologically informed approach that promotes resilience against future disasters by enhancing connection with the environment.

A recent resurgence has emerged in the medical research on psychedelic plants. Beyond treatment of mental health conditions such as depression and addictions, psychedelic plant medicines may be relational compounds—enhancing connection to others, to the land, and to the environment (Kettner et al., 2019; Watts et al., 2022; Irvine et al., 2023). Indeed, among many traditional uses of plant medicines has been fostering connection to the land and nature through their spiritual inhabitants and teachings, as well enhancing other connections such as to community and ancestors. Such historical use recalls the importance of Indigenous wisdom and traditional ecological knowledge, which represent long-standing relational paradigms. Ecological Medicine suggests the importance of more authentic, reciprocal, and reparative dialog with Indigenous groups to better understand relational knowledge and to guide our own movement toward non-exceptionalist worldviews and incorporation of relationality into human health and stewardship of the Earth. Indigenous nations have long understood a worldview that many healthcare organizations and governments have not yet adopted—that health of the planet, health of diverse species of life, and health of humanity are all inter-connected and inter-dependent.

It is important to note that this consensus process has a number of limitations. The Ecological Medicine Working Group may have been homogeneous in its intellectual beliefs and biases, limiting diversity of viewpoints. There may have been selection bias in choosing the Working Group, as selection methodology was neither systematic nor geared toward optimizing diversity of demographics, academic fields, and views. The modified Delphi methodology may have introduced biases, including biasing consensus toward a majority view. Ecological Medicine itself has limitations, two of which are particularly worth noting. First, there is still a bias toward human health rather than toward zoocentric or phytocentric views, which are outside the scope of this study. Second, as with health care generally, there are significant structural barriers to equitable access to nature contact and to the health interventions discussed here. Discussion of equity and justice is paramount in the future development of this field.

## Conclusion

The Ecological Medicine Working Group has proposed a consensus definition of Ecological Medicine as an inter-connectivity- and relationality-based view of human health, with inter-dependencies between people, other species of life, and the natural environment being integral to understanding health. In contrast to the relational worldviews of Indigenous cultures, current Western conceptualization of human health has moved in parallel with its culture—toward individualist and human exceptionalist frameworks. Ecological Medicine represents a paradigm shift in the understanding of health—offering clinical interventions, pathways for education and research, and guiding new approaches for healthcare organizations and governmental policies. Consensus priorities include research, practice, and education in an array of practices that foster connection to self (such as contemplative practices), connection to others (such as social and group practices), and connection to nature (such as interventions to foster human-plant relationships, foster human-animal relationships, and connect to the natural environment). Encouraging trans-disciplinary development and collaborative growth of the field would accelerate integration into clinical practice, research collaboration and funding, academic and public acceptance and visibility, and momentum toward advocacy and policy change. The inseparability of human health and planetary health calls for reciprocity in the healthcare system and planetary stewardship; reciprocity is not only an important basis of connecting and health-enhancing interventions but can be a health-enhancing behavior in itself.
